# Characterization of the first knock-out *aldh7a1* zebrafish model for pyridoxine-dependent epilepsy using CRISPR-Cas9 technology

**DOI:** 10.1371/journal.pone.0186645

**Published:** 2017-10-20

**Authors:** Nikita Zabinyakov, Garrett Bullivant, Feng Cao, Matilde Fernandez Ojeda, Zheng Ping Jia, Xiao-Yan Wen, James J. Dowling, Gajja S. Salomons, Saadet Mercimek-Andrews

**Affiliations:** 1 Genetics and Genome Biology Program, Research Institute, The Hospital for Sick Children, Toronto, Canada; 2 Division of Clinical and Metabolic Genetics, Department of Pediatrics, University of Toronto, Toronto, Ontario, Canada; 3 Neurosciences and Mental Health Program, Research Institute, The Hospital for Sick Children, Toronto, Ontario, Canada; 4 Department of Physiology, University of Toronto, Toronto, Canada; 5 Metabolic Laboratory, Department of Clinical Chemistry, VU University Medical Center, Amsterdam, Neuroscience Amsterdam, The Netherlands; 6 Zebrafish Centre for Advanced Drug Discovery & Keenan Research Centre for Biomedical Science, Li Ka Shing Knowledge Institute, St. Michael's Hospital, Toronto, Ontario, Canada; 7 Division of Neurology, Department of Pediatrics, University of Toronto, Toronto, Ontario, Canada; 8 Department of Molecular Genetics, University of Toronto, Toronto, Ontario, Canada; 9 Neuroscience Campus, Amsterdam, The Netherlands; 10 Institute of Medical Sciences, University of Toronto, Toronto, Ontario, Canada; Alberta Children's Hospital, CANADA

## Abstract

Pyridoxine dependent epilepsy (PDE) is caused by likely pathogenic variants in *ALDH7A1 (*PDE-*ALDH7A1*) and inherited autosomal recessively. Neurotoxic alpha-amino adipic semialdehyde (alpha-AASA), piperideine 6-carboxylate and pipecolic acid accumulate in body fluids. Neonatal or infantile onset seizures refractory to anti-epileptic medications are clinical features. Treatment with pyridoxine, arginine and lysine-restricted diet does not normalize neurodevelopmental outcome or accumulation of neurotoxic metabolites. There is no animal model for high throughput drug screening. For this reason, we developed and characterized the first knock-out *aldh7a1* zebrafish model using CRISPR-Cas9 technology. Zebrafish *aldh7a1* mutants were generated by using a vector free method of CRISPR-Cas9 mutagenesis. Genotype analysis of *aldh7a1* knock-out zebrafish was performed by high resolution melt analysis, direct sequencing and QIAxcel system. Electroencephalogram was performed. Alpha-AASA, piperideine 6-carboxylate and pipecolic acid, were measured by liquid chromatography-tandem mass spectrometry. Our knock-out *aldh7a1* zebrafish has homozygous 5 base pair (bp) mutation in *ALDH7A1*. Knock-out *aldh7a1* embryos have spontaneous rapid increase in locomotion and a rapid circling swim behavior earliest 8-day post fertilization (dpf). Electroencephalogram revealed large amplitude spike discharges compared to wild type. Knock-out *aldh7a1* embryos have elevated alpha-AASA, piperideine 6-carboxylate and pipecolic acid compared to wild type embryos at 3 dpf. Knock-out *aldh7a1* embryos showed no aldh7a1 protein by western blot compared to wild type. Our knock-out *aldh7a1* zebrafish is a well characterized model for large-scale drug screening using behavioral and biochemical features and accurately recapitulates the human PDE-*ALDH7A1* disease.

## Introduction

Pyridoxine dependent epilepsy (PDE) (OMIM#266100) caused by likely pathogenic variants in *ALDH7A1* (PDE-*ALDH7A1*) (GenBank accession nos: GI319655560 GI320202963 and GI319655559) is an autosomal recessively inherited neurometabolic disease [1,2]. *ALDH7A1* encodes alpha-aminoadipic semialdehyde dehydrogenase (EC 1.2.1.31) in the lysine catabolic pathway and the enzyme deficiency leads to accumulation of alpha-aminoadipic acid semialdehyde (alpha-AASA) and piperideine 6-carboxylate (P6C), the latter inactivates pyridoxal-5-phosphate [2]. Pipecolic acid (PA) elevations in plasma and cerebrospinal fluid (CSF) were reported in PDE-*ALDH7A1* patients as a secondary biomarker [3].

Neonatal or early infantile onset seizures refractory to anti-epileptic medications and responsive to pyridoxine is the characteristic phenotype of PDE-*ALDH7A1* patients [4]. Accumulation of alpha-AASA, P6C and PA in body fluids is the characteristic biochemical feature of PDE-*ALDH7A1* [2,5]. Treatment consists of pyridoxine, lysine-restricted diet and arginine to treat seizures and decrease accumulation of CSF alpha-AASA, P6C and PA to improve neurodevelopmental outcome [4,6–10]. However, this treatment was not successful to normalize accumulation of CSF alpha-AASA, P6C and PA or neurodevelopmental outcome in any of the patients so far [6,7,9,10].

To the best of our knowledge, there is no animal model to perform drug screening for this disease. An ideal animal model will also shed light to pathophysiology of PDE-*ALDH7A1* and allow us to examine the neurotoxic effects of alpha-AASA, P6C and PA accumulation in the central nervous system. An ideal model system is the zebrafish due to its utility for nonbiased large-scale drug screening. For this reason, we initiated the characterization of the first zebrafish model of PDE-*ALDH7A1*. We created an *aldh7a1* knockout line using CRISPR-Cas9 that mirrors the phenotypic and biochemical features of the human PDE-*ALDH7A1* disease. Our *aldh7a1* zebrafish is an ideal model for future drug discovery for this severe neurodegenerative disorder.

## Materials and methods

Zebrafish were housed, maintained and bred at the Zebrafish Core Facility. The study was approved by Institutional Animal Care Committee and Use Ethics Board Committee Ethics Board (ACC#41617), Research Institute, The Hospital for Sick Children. This study was carried out in strict accordance with the approved conditions to ameliorate animal suffering.

### *aldh7a1* knock-out zebrafish using CRISPR-Cas9 technology

Zebrafish *aldh7a1* mutants were generated by the Institutional Zebrafish Genetics and Disease Models Core Facility using a vector free method of CRISPR-Cas9 mutagenesis modified from previous report [11]. The program Chopchop (https://chopchop.rc.fas.harvard.edu/) was used to design a guide RNA (gRNA) target site in exon 4 containing a 20-base pair (bp) target sequence (GGTACCTGCTCCAAAGAGAG). The gRNA along with Cas9RNA were injected into 50–100 wild type one-cell stage embryos. Cas9 cutting was confirmed via high-resolution melt (HRM) analysis. The DNA used in HRM was extracted from 21 individual injected embryos and 3 un-injected embryos. The DNA was mixed with 1x PCR buffer and 1 μg proteinase k and incubated for 50 minutes at 55°C followed by a 10-minute incubation at 98°C. HRM analysis was completed on a Roche Lightcycler96 using Roche 2x HRM master mix (04909631001) according to the manufactures instructions. The annealing temperature was 62°C, the final MgCl2 concentration was 3.0 mM. Forward-primer sequence was GCAAAAACAATGCTGACTAATGC and reverse primer sequence was CTGCCGAGAGCTTTGATCTTC for a 114-bp product. Sexually mature F0 adults were outcrossed to wild type. Heterozygous F1 embryos were screened using HRM analysis to identify F0 fish with germline mutations. Heterozygous F1 embryos were identified using DNA sample extracted from clipped tails and HRM analysis. Briefly, DNA samples in 100 μL of 50 mM NaOH were heated at 98° for 20 minutes. 100 μL of 1M Tris pH 8.0 was added. The PCR conditions included: 95°C for 5 minutes, 34 cycles of 95°C for 30 seconds, 59°C for 30 seconds, 72°C for 45 seconds and a final extension time at 72°C for 5 minutes. The following primers were used Forward-TGTGTTATTTAGGTACCTGCTCCA and Reverse-AGCCTCTCCAATCTGTCGAAC.

F2 embryos generated from a single female and male were sacrificed at 3 days’ post fertilization (dpf) and one half was used for DNA extraction and genotyping and one half was used for alpha-AASA, P6C and PA metabolite measurements. To analyze behavior, we applied two methods including 1) F2 embryos from 2–3 females and males were analyzed under light microscope for their behavioral analysis between 3–6 dpf before being sacrificed for genotyping prior to feeding in the system; 2) F2 embryos from a single male and female were analyzed under light microscope for their behavioral analysis between 8–12 dpf and sacrificed for genotyping. F2 embryos from a single male and female were allowed to grow into adulthood for the monitoring of the survival and sacrificed at 20 dpf for genotyping.

### Genotype analysis of *aldh7a1* knock-out zebrafish

#### HRM analysis

DNA was extracted from embryos using 1xPCR buffer and 1 μg proteinase k. Samples were incubated for 50 minutes at 55°C and for 10 minutes at 98°C. HRM analysis was completed on a Roche Lightcycler96 using Roche 2x HRM master mix (04909631001) following the manufacturer’s instructions. The following primers were used Forward-TGTGTTATTTAGGTACCTGCTCCA and Reverse-AGCCTCTCCAATCTGTCGAAC.

The final MgCl_2_ concentration was 3.0 mM. The PCR conditions for HRM included: a pre-incubation step at 95°C for 5 minutes, 45 cycles of 3 step amplification including 95°C for 10 seconds, 64°C for 10 seconds, 72°C for 10 seconds, a HRM step including 95°C for 60 seconds, 40°C for 60 seconds, 65°C for 1 seconds, and 97°C for 1 seconds, and a final cooling step at 37°C. Lightcyler96 software was used for data analysis.

#### Direct sequencing and QIAxcel system

DNA of one hundred zebrafish were isolated. A 159 bp region was amplified by PCR containing the target site of CRISPR-Cas9 resulting in an amplicon of 159 (wild type) or 154 bp (mutant DNA containing 5 bp deletion) nucleotides. The following primers containing a Forward-M13 tail: GTAAAACGACGGCCAGACAATGCTGACTAATGCTATGTGTT and Reverse-CAGGAAACAGCTATGAAATGCAAACTTACTAAGCTGCCG were used. The amplified PCR products were analyzed to determine the size by QIAxcel system. Electropherogram and gel images were generated to identify which zebrafish were homozygous for 5bp deletion. Presence of 5bp deletion mutation was confirmed by direct Sanger sequencing analysis using Applied 3130XL.

### Electroencephalogram (EEG) of knock-out *aldh7a1* zebrafish

EEG recordings of zebrafish embryos were performed according to previously reported method with a slight modification [12]. Low melting 1.2% agarose was prepared within recording media solution (1mM NaCl, 2.9mM KCl, 10mM HEPES, 1.2mM MgCl2, 10mM Dextrose, 2.1mM CaCl2). 0.02% tricaine was used to anesthetize embryos (Sigma Aldrich). Alpha-bungarotoxine (1mg/ml) (Tocris Bioscience, Cedarlane, Burlington, Canada) was used as paralytic agent to reduce movement artefacts. The agarose block was immersed in recording media solution. A microelectrode (approximately 1μm diameter, 2–7 MΩ) was inserted into the amplifier head stage, which was mounted on a micromanipulator. Microelectrodes were fabricated from a 1.5mm OD borosilicate glass, and pulled into two needles with a two-step Narishige puller. The microelectrode was back loaded with recording media solution using a 1 ml syringe with a Corning syringe filter (0.2 μm) and 28 gauge MicroFil filament (World Precision Instruments) attached. Electrical activity was captured with an Axopatch 200B (Axon Instrument) patch clamp amplifier in current clamp mode. Data was collected in pClamp 8 software (Molecular Devices, USA) using gap-free acquisition mode, sampling at 10KHz, gain at 100 mV/pA. A recording chamber and electrophysiology rig were used to stabilize embryos. EEG were analyzed using number of events, duration of each event and amplitude of each event for a single embryo. Average for duration and amplitude were calculated for each embryo. Mann-Whitney *U* test was applied for statistical analysis of the results.

Wild type embryos were treated with 15 mM of pentylenetetrazole (SigmaAldrich, St-Louis Missouri, US) to induce seizure like locomotor behavior during EEG recording [12–14]. Seizure like locomotor behavior was treated with 100 μM diazepam (Sandoz, Boucherville, Canada) and 100 μM phenobarbital in wild type and knock-out *aldh7a1* zebrafish [13]. Knock-out *aldh7a1* zebrafish was treated with 25 mM pyridoxine (SigmaAldrich, St-Louis Missouri, US).

### Behavioral analysis for knock-out *aldh7a1* zebrafish

Seizure-like locomotor behavior was analyzed using previously reported patterns provoked by pentylenetetrazole including: stage I: dramatically increased swimming speed/activity; stage II: rapid motion circular swimming; and stage III: loss of posture and loss of motion for 1–3 seconds [13,14]. The Viewpoint Videotrack for Zebrafish^TM^ system (Version 3.22 with background subtraction, Viewpoint, Québec, Canada) was used to monitor seizure-like locomotor behavior in *aldh7a1* homozygous, heterozygous and wild type zebrafish embryos as described previously at 10 dpf for one hour [14]. Plots were analyzed for distance traveled (in millimeters), total time spend and velocity. ANOVA was used for statistical analysis of the results with calculated standard error of the mean.

### Measurement of alpha-AASA, P6C and PA in knock-out *aldh7a1* zebrafish by liquid chromatography-tandem mass spectrometry (LC-MS-MS)

Embryos were stored at -80°C until measurement of alpha-AASA, P6C and PA. Embryos were kept on ice and lysed using a pestle and mini-homogenizer in 100 μL of cell lysis buffer: 1X RIPA buffer, Pierce phosphatase inhibitor mini tablet (ThermoScientific, Illinoi, US), and complete Mini EDTA-free protease inhibitor cocktail tablet (Roches Diagnostics, Indianapolis, US). Once homogenized, the homogenates were placed on ice for 10 min to complete lysis. The lysates were then centrifuged at 13000 rpm for 10 min at 4°C. Total protein was measured using the DC Protein Assay (BioRad, California, US). Samples were prepared according to a previously reported method with slight modifications [5]. 80 μL of embryos lysate were pipetted in 1.5 mL Eppendorf tubes. Internal standards (200 ng d9-PA, and 200 ng d3-alpha-AAA) and 200 μL acetonitrile were added and samples were mixed by vortex and centrifuged at 20,000g for 10 minutes at 4°C. 200 μL supernatants were transferred into a set of 1 mL concal tubes and combined with 50 μL of borate buffer (0.4M, pH 8.5) and 20 μL of 100 mM FMOC-Cl in acetone. The mixture was stirred for 11 minutes to form FMOC-derivatives. The reaction mixture was cleaned by liquid-liquid extraction with pentane (3 times 2 mL). The upper pentane layer was discarded and the lower layer was transferred into 200 μL inserts for LC-MS-MS analysis.

The measurement of the analytes was performed using liquid chromatography-tandem mass spectrometry using an Infinity 1290 LC series by gradient using a Phenomenex Kinetex column (2.6μm XB-C18, 50 x 3.0 mm) with mobile phase A (90/10 water/acetonitrile + 10 mM ammonium formate pH3.2), and B (10/90 water/acetonitrile + 10 mM ammonium formate pH3.2) at a flow rate of 0.4 mL/minute. Sample injection volume was 1 μL. The total run time was 10 minutes. Tandem mass spectrometry was carried out on an ABSciex QTrap5500 mass spectrometer operated in positive ESI (Electro Spray Ionization) ion mode. The following mass transitions were monitored for MRM acquisition of FMOC-derivatives of compounds: m/z 374.2->263.3 for PA-FMOC, 350.2->179.2 for P6C-FMOC, 390.21 -> 263.2 for α-AASA and 383.2 -> 263.3 for d9-PA-FMOC, 409.2->263.3 for d9-AAA-FMOC.

### aldh7a1 protein analysis by western blot in knock-out *aldh7a1* zebrafish

Lysates of embryos and total protein measurement were prepared with the same methods described in the subsection “Measurement of alpha-AASA, P6C and PA in knock-out *aldh7a1* zebrafish by liquid chromatography-tandem mass spectrometry (LC-MS-MS)”. Lysates were boiled with loading buffer for 10 min at 95°C and loaded on a SDS-polyacrylamide gel. Immunodetection was achieved using a zebrafish aldh7a1 antibody (GenScript, New Jersey, US), B-actin (Cell Signaling, Massachusetts, US) and chemiluminescence (BioRad, California, US).

## Results

### Phenotype, locomotor behavior and genotype analysis of *aldh7a1* knock-out zebrafish using CRISPR-Cas9 technology

There is a single copy of *aldh7a1* in zebrafish. gRNA and Cas9 RNA were injected into 1 cell stage embryos. The F0 embryos were grown to adulthood. Using HRM analysis on these F0 adult zebrafish, we identified two zebrafish carrying four or more mutations. These chimeric zebrafish adults were outcrossed to wild type AB zebrafish. The resulting F1 offspring were raised to adulthood. Screening by both HRM and Sanger sequencing, we identified F1 carriers with the following mutations: a 5bp deletion (AGAGG), an 11bp deletion (GAGAGGGGAAA), and a 21bp insertion (AAATTGTTCGACAATTTTTGA). We incrossed F1 carriers with the 5bp deletion (which is predicted to result in an early stop codon) to generate the F2 generation zebrafish. Those adult F1 carriers with the 5bp deletion were outcrossed to wild type to generate second generation and second generation adult F1 carriers with the 5bp deletion were outcrossed to wild type to generate third generation heterozygous 5bp deletion females and males to eliminate off target effects. These heterozygous females and males were used for characterization of knock-out 5 bp deletion *aldh7a1* zebrafish.

**Fig 1:**
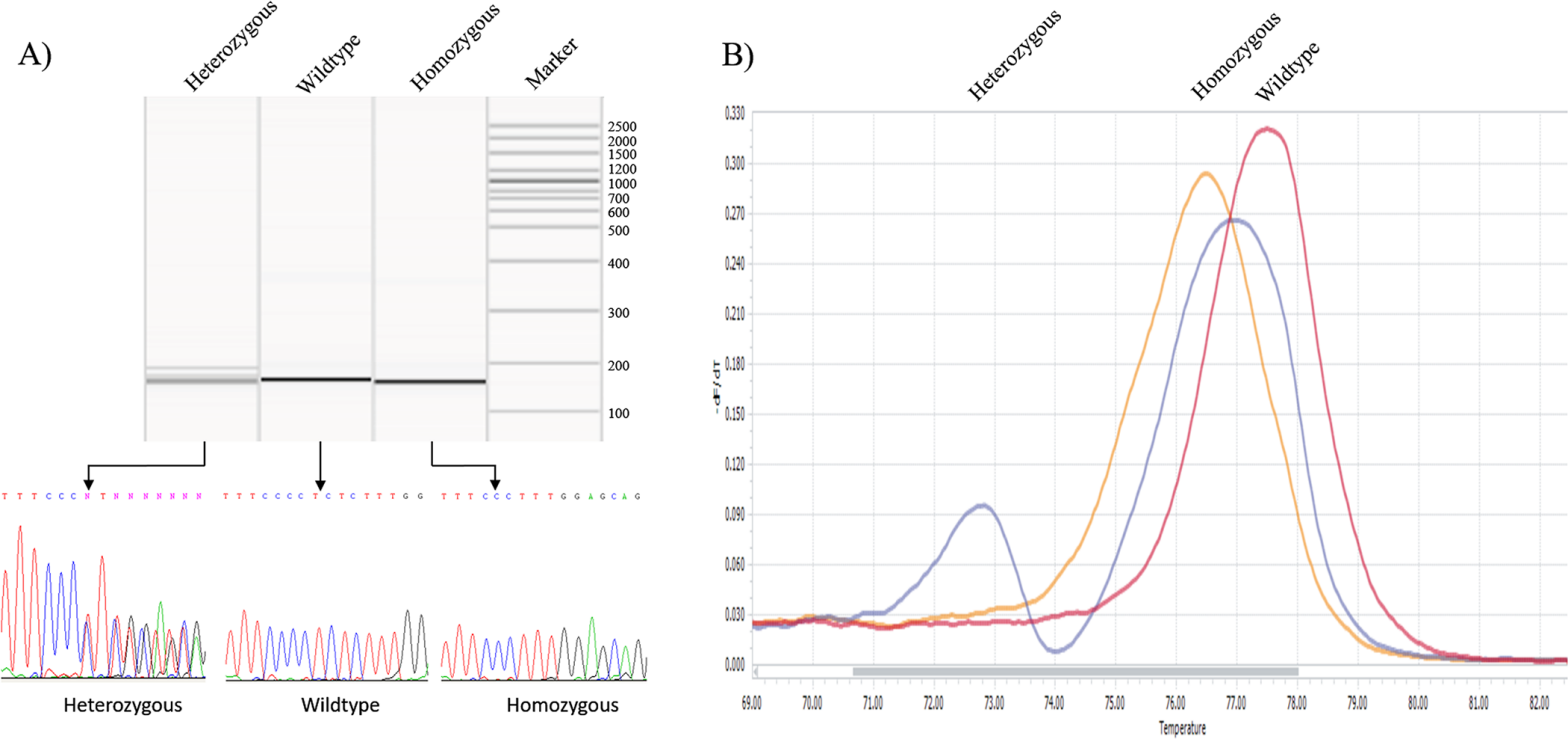
QIAxcel system, Sanger sequencing and HRM analysis results. A) Gel image produced by QIAxcel system revealing wild type *aldh7a1* sequence, as well as the heterozygous and homozygous 5bp deletion. The DNA sequences shown in the lower panel reveal the complement reverse sequences. The broken line indicates the nucleotides that were deleted by CRISPR/Cas9 in the homozygous fish. B) The HRM melting plots are displayed. The heterozygous sequence is identified by the double peak. The homozygous sequence is identified by a peak at 76.5 degrees Celsius, and the wild type sequence is identified by a peak at 77.5 degrees Celsius.

Using HRM analysis the heterozygous zebrafish were identified by the double peak. The homozygous and wild type zebrafish had a single peak with distinct melting temperatures. The peaks with different melting temperatures were cross referenced with direct sequencing results to allow us to call the higher temperature peaks as wild type and lower temperature peaks as homozygous (Fig 1). Using direct sequencing and QIAxcel system, we identified a single 154 bp PCR amplicon fragment as homozygous, which was confirmed by Sanger sequencing (Fig 1). We sacrificed 67 embryos from a single heterozygous 5 bp deletion female and male at 3 dpf. We identified 16 homozygous, 36 heterozygous and 15 wild type embryos.

**Fig 2:**
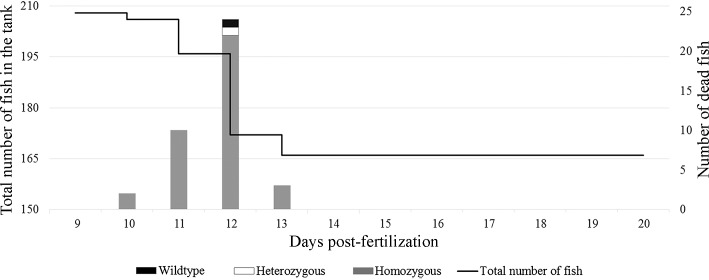
Daily survival of heterozygous 5 bp deletion male and female zebrafish embryos. Y axis to the left indicates total number of embryos in the tank. Y axis to the right indicates the number of dead embryos genotyped and presented as a histogram. X axis shows dpf.

Embryos with various phenotypic features to genotype were collected including 1) decreased pigmentation; 2) small compared to peers; 3) curved tail; 4) edema; 5) curvature on the back. None of those phenotypes were homozygous for 5bp deletion. Embryos from heterozygous 5 bp deletion females and males were allowed to grow into adulthood and genotyped using DNA samples acquired by fin clipping. Genotyping of 57 surviving zebrafish adults revealed only wild type or heterozygous adults, confirming that the 5 bp deletion homozygous *aldh7a1* zebrafish did not survive into adulthood. Data was generated using one clutch of heterozygous female and male pair. There were 238 eggs and 207 of them were fertilized. We observed embryos daily and collected dead ones for genotyping. Survived zebrafish embryos were sacrificed at 20 dpf for genotyping. Genotyping of 207 zebrafish embryos (including survivors and dead) revealed 39 homozygous (all collected after their death), 115 heterozygous and 53 wild type. There were no survived homozygous embryos at 20 dpf (Fig 2). The survival data is represented in Fig 2. In our various experiments, there were no survived homozygous after 14 dpf.

**Fig 3:**
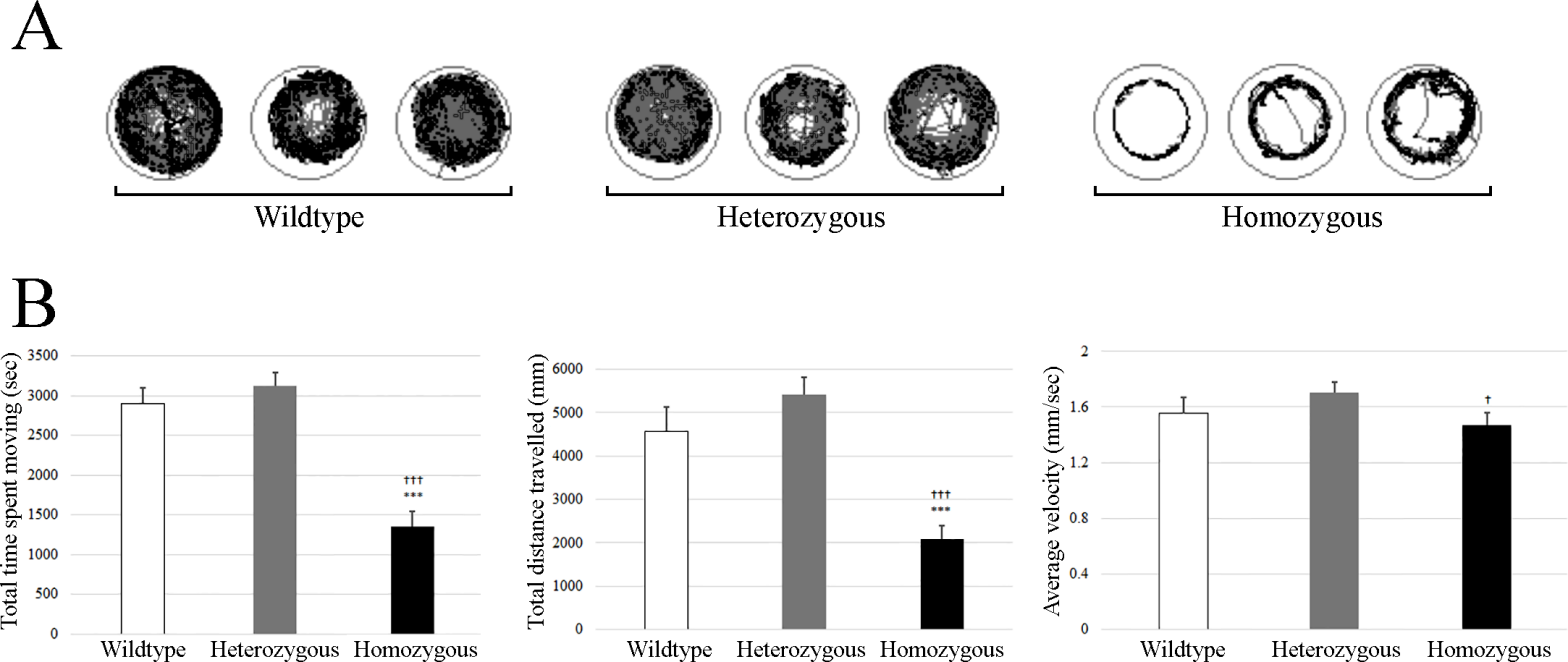
Locomotor behavior of zebrafish embryos using Viewpoint Videotrack for Zebrafish^TM^. A) This represents cumulative plots of the position and velocity of a single knock-out homozygous and heterozygous 5 bp deletion *aldh7a1*embryo and wild type embryo at 10 dpf during one hour of behavioral recording. B) This represents average of total time spent moving (seconds), average of total distance travelled and average of velocity for homozygous (n = 20), heterozygous (n = 20) and wild type (n = 11) embryo at 10 dpf during one hour of behavioral recording. Embryos were placed in individual wells of a flat-bottom 96-well plate and acclimated to the recording chamber before tracking began. One hour of movement data were collected. Data shown are sums of 60-minute (average ± standard error). Error bars shows standard error of the mean (SEM). * is confidence level in comparison with wild type: * is p<0.05, *** is p<0.001. † is confidence level in comparison with heterozygous 10 dpf († - p<0.05, ††† - p<0.001).

We collected 51 embryos at 10 dpf and analyzed their movements using Viewpoint Videotrack for Zebrafish^TM^. The genotyping identified 20 homozygous and 20 heterozygous for 5 bp deletion and 11 wild type embryos at age of 10 dpf. Based on their genotype, we grouped them to compare their recorded locomotor behavior and found statistically significant difference between homozygous and wild type for total time spent moving and total distance travelled. Comparison of total time spent moving, total distance travelled and average total velocity for homozygous, heterozygous and wild type is shown in Fig 3.

All embryos from incrossed heterozygous 5 bp deletion females and males were placed in system water for feeding from 5 dpf according to our institutional zebrafish feeding guidelines. They were analyzed daily starting from 6 dpf to identify behavioral phenotype, genotype and survival. Between 10 to 13 dpf, all embryos with a rapid circling swim behavior followed by loss of posture or all dead embryos were homozygous for 5bp deletion (S1 Video). Phenotypic analysis of homozygous 5 bp deletion *aldh7a1* embryos did not show any specific physical characteristics to differentiate homozygous from wild type or heterozygous.

### Electroencephalogram (EEG) of knock-out homozygous 5 bp deletion *aldh7a1* zebrafish

**Fig 4:**
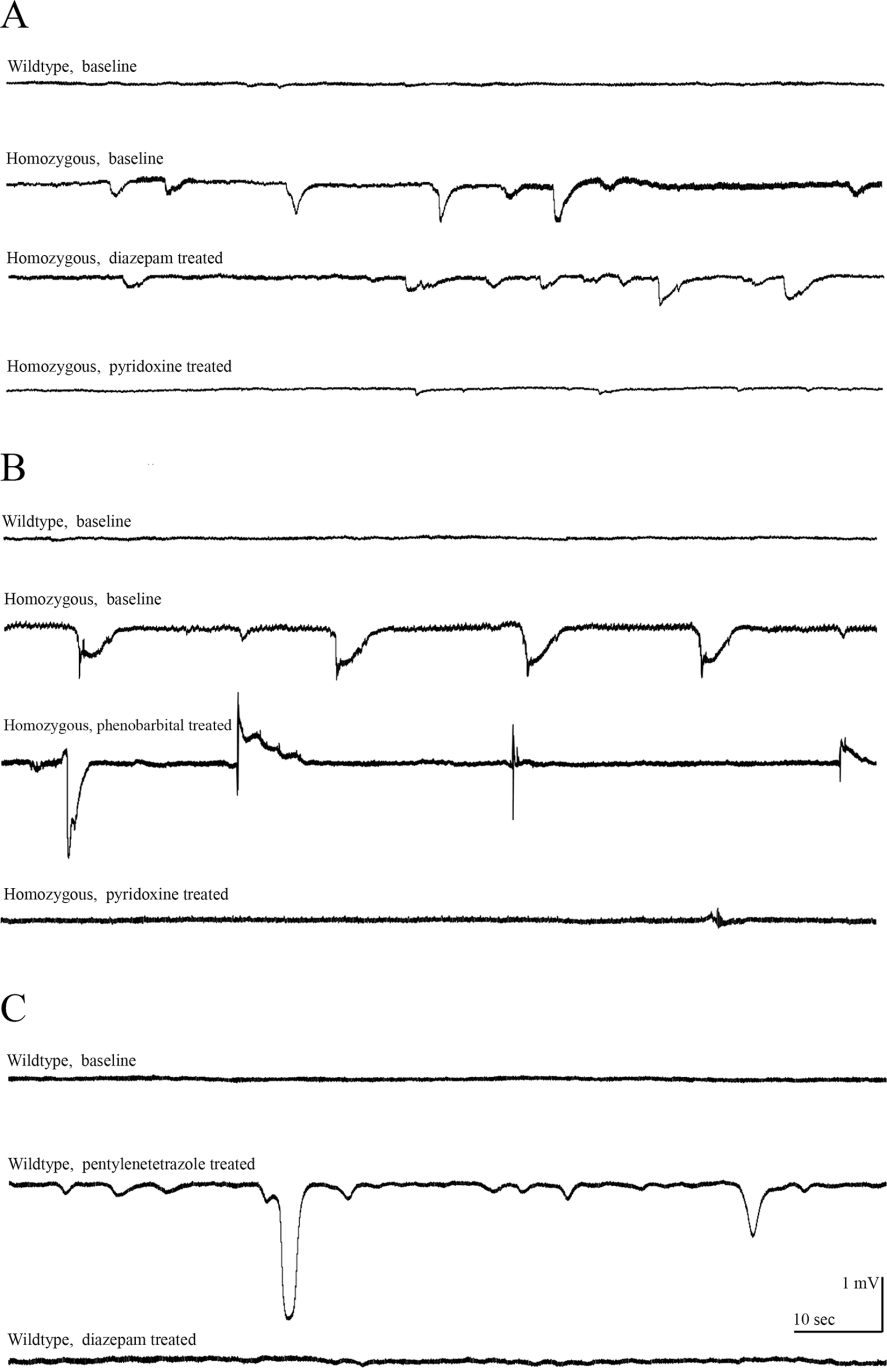
EEG results of knock-out homozygous *aldh7a1* embryos and wild type embryos at 9 dpf. A) This shows spike discharges in a homozygous with no response to 100 μM diazepam, but almost normalization of EEG on 25 mM pyridoxine compared to wild type. B) This shows another homozygous with no response to phenobarbital, but almost normalization of EEG on pyridoxine (25 mM) compared to wild type. C) This shows normal EEG with spikes on pentylenetetrazole, and normalization of EEG on diazepam.

**Fig 5:**
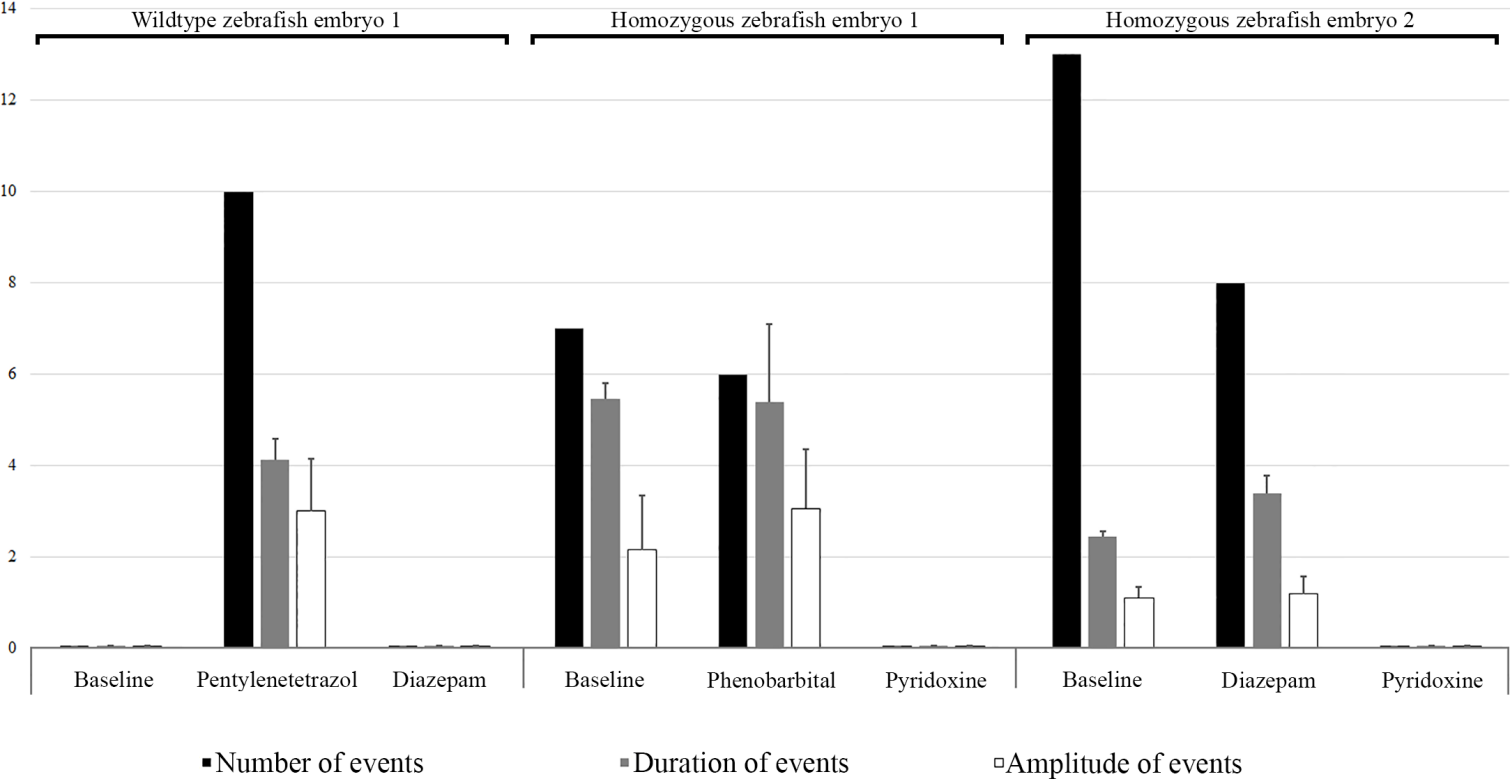
EEG results of two knock-out homozygous *aldh7a1* embryos and wild type embryo at 9 dpf. The results represented as number of events (per 300 seconds), duration of each event (seconds) and amplitude (mV) of each event as baseline and on treatment. Each event was considered as a single spike discharge of the EEG recording. Right panel reports events in a knock-out homozygous *aldh7a1* embryo, their treatment with 100 μM diazepam and followed by 25 mM pyridoxine treatment. The middle panel shows events in another knock-out homozygous *aldh7a1* embryo, their treatment with 100 μM diazepam and followed by 25 mM pyridoxine treatment. The left panel reports events in a wild type on 15 mM pentylenetetrazole to produce spikes and treat them with 100 μM diazepam. Error bars of are standard error of the mean (SEM).

We incrossed heterozygous 5 bp deletion females and males to perform EEG. Embryos with rapid circling swim behavior followed by loss of posture were selected for EEG at 9 dpf together with age matched wild type embryos. A single embryo was anesthetized with 0.02% tricaine and alpha-bungarotoxine in 1.2% low melting agarose then transferred to a recording chamber submerged with recording media. Embryo was visualized using a 4 times microscope and a microelectrode was settled in forebrain for recording of electrical activity in current clamp mode. Large amplitude spike discharges were observed in homozygous 5 bp deletion *aldh7a1* embryo, which was confirmed by genotype analysis after completion of EEG recording. During EEG recording of each zebrafish embryo individually, one homozygous 5 bp deletion *aldh7a1* embryo was treated with 100 μM diazepam with no improvements in large amplitude spike discharges, which improved with 25 mM pyridoxine (Fig 4A). During EEG recording, another homozygous 5 bp deletion *aldh7a1* single embryo was treated with 100 μM phenobarbital with no improvements in spike discharges, which improved with 25 mM pyridoxine (Fig 4B). We also treated a single wild type embryo with 15 mM pentylenetetrazole to induce spikes and treated embryo with 100 μM diazepam afterwards to improve spikes (Fig 4C). To show effects of the treatment, we analyzed EEG of two different homozygous 5 bp deletion *aldh7a1* embryos and a wild type embryo for number of events, duration of each event and amplitude of each event as baseline and on treatment and average for each parameter is given in Fig 5. Our results confirm the previously reported responses to pentylenetetrazole and diazepam [13].

### Biochemical characterization of knock-out *aldh7a1* zebrafish

**Fig 6:**
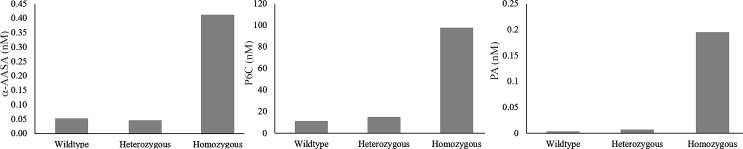
Metabolite measurement of alpha-AASA, P6C and PA. 3dpf zebrafish embryo lysates (n = 15) were analyzed by liquid chromatography tandem mass spectrometry for homozygous and heterozygous for 5 bp deletion *aldh7a1* embryos and wild-type embryos. These show elevated levels of alpha-AASA, P6C and PA compared to wild type.

Knock-out homozygous 5 bp deletion *aldh7a1* embryos had 8 times elevation of alpha-AASA, 9 times elevation of P6C and 55 times elevation of PA compared to wild-type embryos at 3 dpf (Fig 6). Accumulation of alpha-AASA, P6C and PA was evident as early as 24–48 hours of hatching from eggs to embryo following breeding of heterozygous females and males.

### aldh7a1 protein by western blot analysis in knock-out *aldh7a1* zebrafish

**Fig 7:**
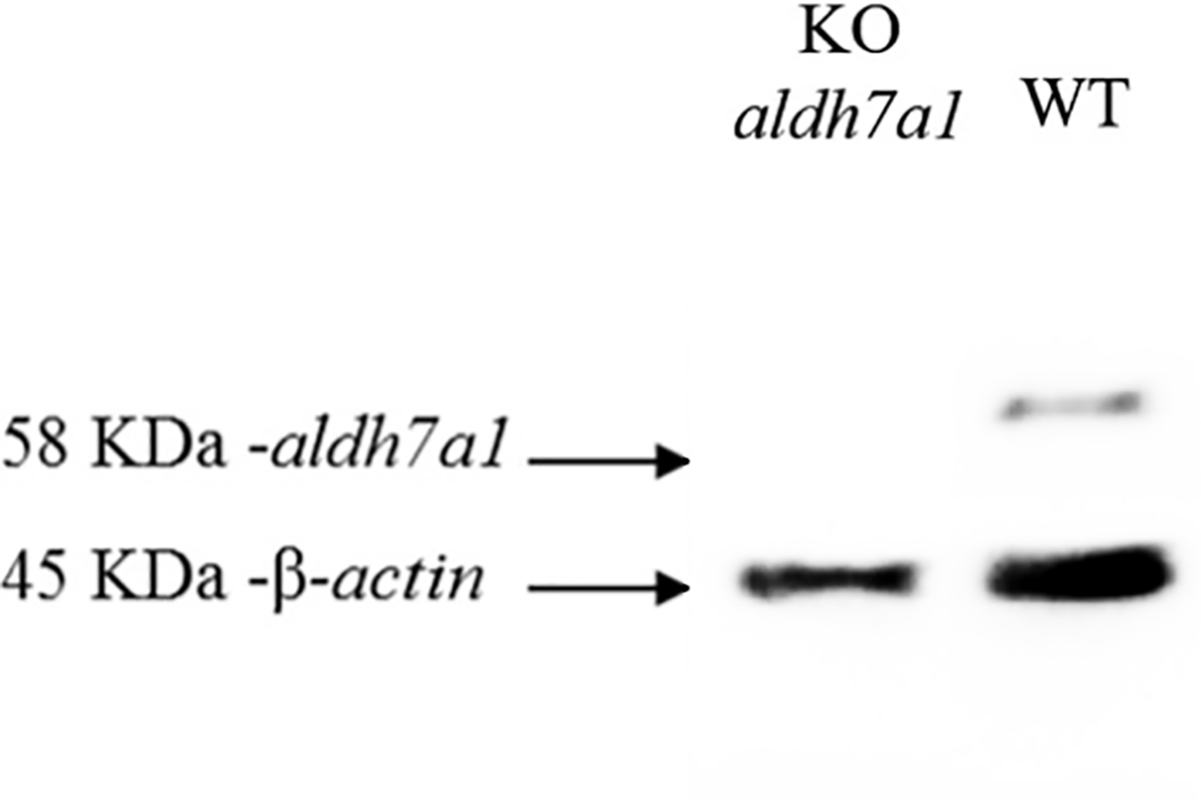
Western blot. Lysates of knock-out homozygous *aldh7a1* zebrafish embryos (at 8 dpf) western blot analysis showing no aldh7a1 protein compared to wild-type.

Knock-out homozygous 5 bp deletion *aldh7a1* embryos showed no aldh7a1 protein compared to wild type by western blot analysis (Fig 7). No aldh7a1 expression in homozygous zebrafish embryos confirms that 5 bp deletion resulted in loss of function of aldh7a1 protein.

## Discussion

We report behavioral and biochemical characterization of knock-out homozygous 5 bp deletion *aldh7a1* zebrafish model using CRISPR-Cas9 technology for the human PDE-*ALDH7A1* for the first time. Our knock-out *aldh7a1* zebrafish model showed spontaneous rapid increase in locomotion and a rapid circling swim behavior followed by loss of posture as seizure-like locomotor behavior as early as 8 dpf, which resulted in death shortly after this seizure-like locomotor behavior. EEG recording of embryos with seizure-like locomotor behavior revealed large amplitude spike discharges. Those large amplitude spike discharges did not normalize on anti-seizure medications, but normalized on pyridoxine treatment during EEG recording. Genotyping confirmed that those embryos were knock-out homozygous 5 bp deletion *aldh7a1* zebrafish embryos. Neurotoxic alpha-AASA, P6C, PA metabolites were moderate to markedly elevated in our model. Western blot analysis of knock-out homozygous 5 bp deletion *aldh7a1* embryos showed no protein expression confirming the loss of function of *ALDH7A1*. These studies confirm that our knock-out homozygous 5 bp deletion *aldh7a1* zebrafish model is an excellent model for large-scale drug screening using behavioral, electrophysiological and biochemical features and accurately models the human PDE-*ALDH7A1* disease.

Babcock et al [15] reported that knock-down *aldh7a1* zebrafish had small jaw, small eye, decreased pigmentation, curved tail and shorter pectoral fin pointing out anatomical malformations outside of the central nervous system. The aldh7a1 protein was reported as being an important regulator of eye and limb development, but the authors did not evaluate seizures in their knockdown *aldh7a1* zebrafish model [15]. We did not identify any phenotypic features to differentiate homozygous from heterozygous or wild type embryos in our knock-out model. Seizure-like locomotor behavior was the only feature to differentiate homozygous from heterozygous or wild type embryos. We think that previously reported phenotypic features of knock-down *aldh7a1* zebrafish model are not *aldh7a1* gene specific in zebrafish. We had ample opportunity to study phenotypic features of our *aldh7a1* zebrafish model using knock-out model and made an important discovery for the future zebrafish research.

The current treatment of PDE-*ALDH7A1* includes pyridoxine, lysine-restricted diet and arginine [6,7,9,10,16,17]. This combined therapy results in some clinical and biochemical improvements, but patients do not achieve normal neurodevelopmental outcome or normalization of the CSF alpha-AASA levels [6,7,9,10,16,17]. Lysine-restricted diet is cumbersome due to 1) none-palatable taste of medical food; 2) very strict protein restriction causing decreased quality of life of patients and families as well as compliance problems; 3) requirement of metabolic dietician to apply lysine-restricted diet; 4) risks of protein malnutrition and cerebral serotonin deficiency [16]. Lysine is an essential amino acid and is required for sufficient growth, carnitine synthesis, calcium absorption and formation of collagen and elastin. The minimum required daily lysine intake is 35 mg kg^-1^ day^-1^ in childhood [18]. The lowest recommended lysine restriction is at or above 35 mg kg^-1^ day^-1^ in patients with PDE-*ALDH7A1* [17]. Unfortunately, it is not known if this recommended level of lysine restriction is safe, as there are no studies looking at bone mineral density or cardiovascular system on lysine-restricted diet. It is not clear, if lysine-restricted diet will increase long-term morbidity by increasing risk of osteoporosis and bone fractures and risk of aneurisms, stroke and coronary artery disease. These barriers necessitate ongoing search to identify new treatment modalities for human PDE-*ALDH7A1* to improve the neurodevelopmental outcome and quality of life of patients. We think that it is timely to use our knock-out *aldh7a1* zebrafish model to facilitate moderate-to-high-throughput drug screening.

There are only a few genetic disorders with well-characterized seizure phenotype in zebrafish model such as *scn1a* and *stxbp1* knockdown zebrafish [19,20]. *scn1a* zebrafish model was used for moderate-to-high throughput drug screening and identified 4 compounds improving seizure-like locomotor behavior [20]. However, there are no biomarkers to measure effectiveness of the treatment in both models, other than improvements in the seizure-like locomotor behavior. To the best of our knowledge, our knock-out *aldh7a1* zebrafish model is the first model, in which we have proven accumulation of disease specific alpha-AASA, P6C and PA metabolites. Our model will allow us to perform moderate-to-high throughput drug screening to asses effectiveness of drugs not only on seizure-like locomotor behavior and survival into adulthood but also normalization of alpha-AASA, P6C and PA accumulation. Our knock-out model will help us to identify the most effective compound to be able to treat human PDE-*ALDH7A1*.

## Conclusions

We report the first animal model of human PDE-*ALDH7A1*. Our knock-out *aldh7a1* zebrafish will serve as an ideal model for seizure-like locomotor behavior, survival into adulthood and biochemical features of this human disease for drug discovery. Our knock-out *aldh7a1* zebrafish is one of the first disease models generated using the newest CRISPR-Cas9 technology. Further studies will allow us to explore disease neuropathogenesis and increase our understanding.

## Supporting information

S1 Video Behavioral phenotype of homozygous 5 bp deletion *aldh7a1* embryo. Video recording of knock-out homozygous 5 bp deletion *aldh7a1* embryo at 10 dpf shows a rapid circling swim behavior followed by loss of posture compared to wild type.
(WMV)
